# Economic Evaluation of Immunisation Programme of 23-Valent Pneumococcal Polysaccharide Vaccine and the Inclusion of 13-Valent Pneumococcal Conjugate Vaccine in the List for Single-Dose Subsidy to the Elderly in Japan

**DOI:** 10.1371/journal.pone.0139140

**Published:** 2015-10-07

**Authors:** Shu-ling Hoshi, Masahide Kondo, Ichiro Okubo

**Affiliations:** 1 Department of Health Care Policy and Health Economics, Faculty of Medicine, University of Tsukuba, Tsukuba, Ibaraki, Japan; 2 Department of Health Care Policy and Health Economics, Faculty of Medicine, University of Tsukuba, Tsukuba, Ibaraki, Japan; 3 Department of Health Care Policy and Health Economics, Faculty of Medicine, University of Tsukuba, Tsukuba, Ibaraki, Japan; Univ. of Texas Health Science Center at San Antonio, UNITED STATES

## Abstract

**Background:**

Currently in Japan, both 23-valent pneumococcal polysaccharide vaccine (PPSV–23) and 13-valent pneumococcal conjugate vaccine (PCV–13) are available for the elderly for the prevention of *S*. *pneumoniae*-related diseases. PPSV–23 was approved in 1988, while the extended use of PCV–13 was approved for adults aged 65 and older in June 2014. Despite these two vaccines being available, the recently launched national immunisation programme for the elderly only subsidised PPSV–23. The framework of the current immunisation programme lasts for five years. The elderly population eligible for the subsidised PPSV–23 shot for the 1st year are those aged 65, 70, 75, 80, 85, 90, 95 and ≥100. While from the 2nd year to the 5th year, those who will age 65, 70, 75, 80, 85, 90, 95 and 100 will receive the same subsidised shot.

**Methods:**

We performed economic evaluations to (1) evaluate the efficiency of alternative strategies of PPSV–23 single-dose immunisation programme, and (2) investigate the efficiency of PCV–13 inclusion in the list for single-dose pneumococcal vaccine immunisation programme. Three alternative strategies were created in this study, namely: (1) current PPSV–23 strategy, (2) 65 to 80 (as “65–80 PPSV–23 strategy”), and (3) 65 and older (as “≥65 PPSV–23 strategy”). We constructed a Markov model depicting the *S*. *pneumoniae*-related disease course pathways. The transition probabilities, utility weights to estimate quality adjusted life year (QALY) and disease treatment costs were either calculated or cited from literature. Cost of per shot of vaccine was ¥8,116 (US$74; US$1 = ¥110) for PPSV–23 and ¥10,776 (US$98) for PCV–13. The model runs for 15 years with one year cycle after immunisation. Discounting was at 3%.

**Results:**

Compared to current PPSV–23 strategy, 65–80 PPSV–23 strategy cost less but gained less, while the incremental cost-effectiveness ratios (ICERs) of ≥65 PPSV–23 strategy was ¥5,025,000 (US$45,682) per QALY gained. PCV–13 inclusion into the list for single-dose subsidy has an ICER of ¥377,000 (US$3,427) per QALY gained regardless of the PCV–13 diffusion level. These ICERs were found to be cost-effective since they are lower than the suggested criterion by WHO of three times GDP (¥11,000,000 or US$113,636 per QALY gained), which is the benchmark used in judging the cost-effectiveness of an immunisation programmne.

**Conclusions:**

The results suggest that switching current PPSV–23 strategy to ≥65 PPSV–23 strategy or including PCV–13 into the list for single-dose subsidy to the elderly in Japan has value for money.

## Introduction

23-valent pneumococcal polysaccharide vaccine (PPSV–23) has been recommended for prevention of invasive pneumococcal disease (IPD) in adults since 1983 [1]. It was the only pneumococcal vaccine available for all adults aged 65 and older until the approval of the extended use of 13-valent pneumococcal conjugate vaccine (PCV–13) for prevention of pneumococcal pneumonia and IPD in adults 50 years and older on December 30, 2011 by US Food and Drug Administration [1]. On August 13, 2014, the Advisory Committee on Immunization Practices of US Centers for Disease Control and Prevention modified the recommendation on pneumococcal vaccine for the elderly. The new recommendation states that *“Both PCV13 and PPSV23 should be routinely administered in series to all adults aged ≥65 years”*, which is based on the results of a randomised placebo-controlled trial showing PCV–13 efficacy against community-acquired pneumonia (CAP) among approximately 85,000 adults aged 65 and older [1]. PPSV–23 and PCV–13 differ in cost, number of serotypes covered, mechanism for immunogenicity, and level of effectiveness, particularly against non-bacteremic pneumococcal pneumonia (NPP).

Currently, in Japan, both PPSV–23 and PCV–13 are available for the elderly for the prevention of *S*. *pneumoniae-*related diseases. PPSV–23 was approved in 1988 [2]. However, only some municipalities coordinated publicly-funded pneumococcal immunisation programmes for the elderly from 2001 through September 2014; vaccine coverage was about 25% [3]. On the other hand, the extended use of PCV–13 in adults aged 65 and older was approved in June 2014. Despite of these two vaccines being available for elderly, the national immunisation programme launched for the elderly aged 65 and older on October 1, 2014 only subsidised PPSV–23. The framework of the current immunisation program lasts for five years. The elderly population eligible for the subsidised PPSV–23 shot for the 1^st^ year are those aged 65, 70, 75, 80, 85, 90, 95 and ≥100. While from the 2^nd^ year to the 5^th^ year, those who will age 65, 70, 75, 80, 85, 90, 95 and 100 will receive the same subsidised shot [4]. Countries, where publicly funded PPSV–23 immunisation programmes for elderly have been launched, set the eligible age to receive a shot of subsidised vaccine as 65 to 80, 65 and older, 70 and older, and so on [1, 5–9]. Due to the limited resources for health care, there is a need to organize an efficient immunisation programme. This study builds upon such need and intends to address such issues by (1) evaluating the efficiency of alternative strategies of PPSV–23 immunisation programmes, and (2) investigating the efficiency of PCV–13 inclusion in the list of single-dose pneumococcal vaccine national immunisation programme.

## Methods

We conducted a cost-effectiveness analysis with Markov modelling from payers’ perspective. We conducted a literature survey to define the alternative immunisation programmes and to construct the model. Studies pertaining to epidemiology and prognosis of relevant diseases caused by *S*. *pneumoniae* in Japan’s setting were accessed from PubMed database, Igaku Chuo Zasshi (Japana Centra Revuo Medicina) database, Ministry of Health, Labour and Welfare (MHLW) Grant System, and annual statistic reports published by the government. Igaku Chuo Zasshi, a Japanese medical bibliographic database, which contains over 10 million citations originating from Japan, comprehensively covers articles published in Japanese-language medical journals. Due to insufficient evidences from Japan, overseas’ reports from PubMed, Medline, The Cochrane Database of Systematic Reviews, Health Technology Assessment database, and The NHS Economic Evaluation Database regarding vaccine effectiveness, utility weights to estimate quality adjusted life year (QALY) and economic evaluation related to vaccines were used instead.

### PPSV–23 programmes and inclusion of PCV–13

The target population of the immunisation programmes to be evaluated are those aged 65 and older in 2014. In evaluating the efficiency of different PPSV–23 immunisation programmes, we set three different strategies with different ages to receive a shot of subsidised vaccine, namely: (1) current PPSV–23 strategy, (2) 65 to 80 (as “65–80 PPSV–23 strategy”), and (3) 65 and older (as “≥65 PPSV–23 strategy”). Age-specific populations were from demographic data [10]. Current PPSV–23 strategy served as a comparator of the other two strategies. In 65–80 PPSV–23 strategy, those who aged over 80 were not eligible to the immunisation programme, which means these individuals will only follow the transition probabilities assigned to the corresponding ages without any vaccine effectiveness on reducing any *S*. *pneumoniae*-related diseases. Vaccine uptake rates were assumed at 50.4% for all strategies, which was the same with the coverage rate of seasonal influenza vaccine in 2013 [11].

In order to investigate the cost-effectiveness of PCV–13 inclusion in the list for single-dose pneumococcal vaccine national immunisation programme, we made variations on the share of PCV–13 between the two pneumococcal vaccines from 10% to 100% with 10% interval, because it is unknown how doctors, vaccinees, and municipalities will choose between PPSV–23 and PCV–13. Ten levels of diffusion of PCV–13 were compared with current PPSV–23 strategy.

Only single-dose subsidy was analysed and not the sequence of PCV–13/PPSV–23 or PPSV–23/PCV–13, this is mainly due to PPSV–23 immunisation being a newly-launched programme in Japan [12, 13]. We reserve the evaluation of the cost-effectiveness of uptaking the two vaccines in the future research so as to delineate and emphasize on the main purpose of the study.

### Markov model

**Fig 1:**
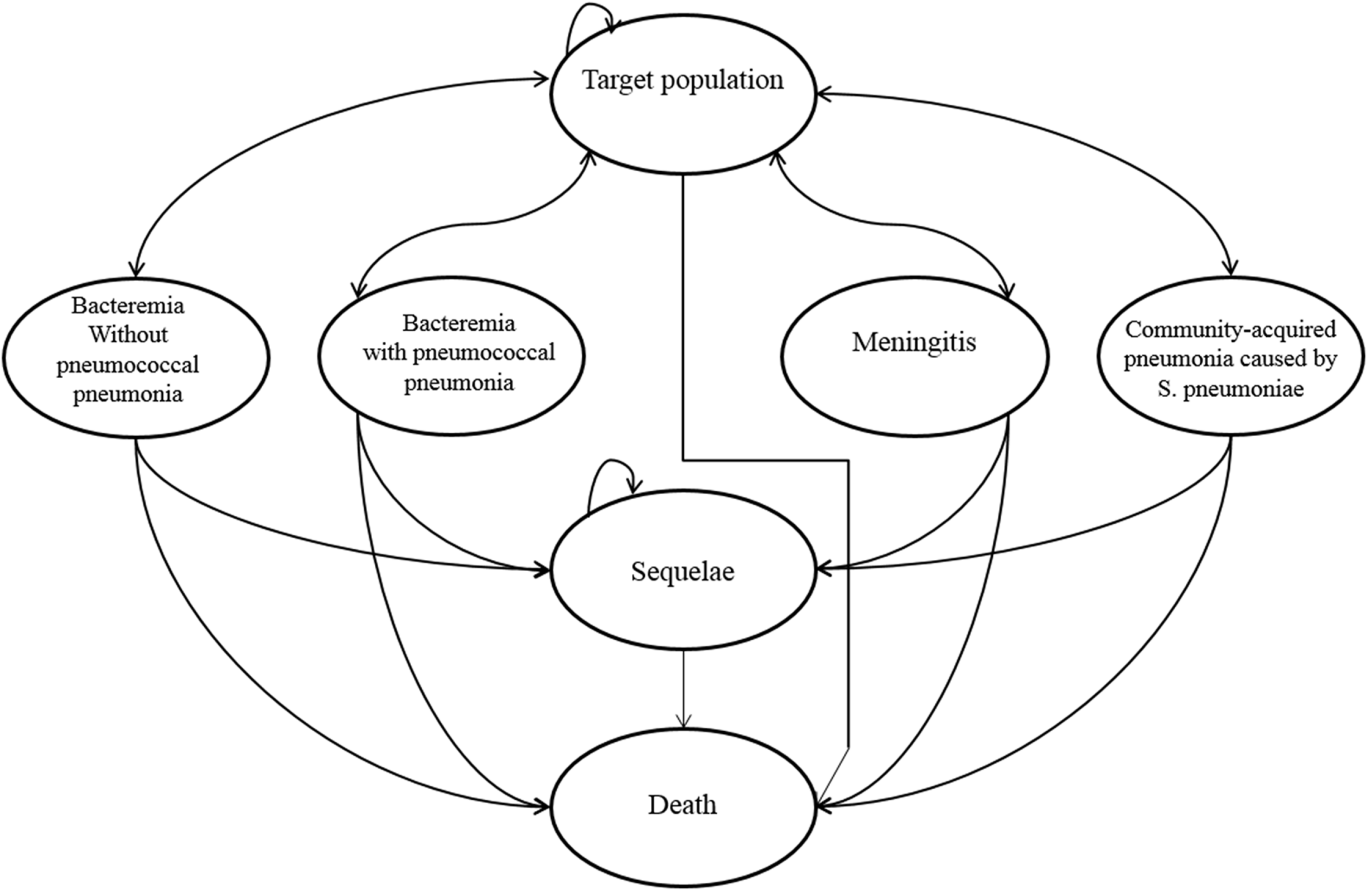
Markov Model.

A Markov model of courses followed by the cohort under consideration was constructed based on epidemiological data, vaccine effectiveness and models from previous studies. Seven mutually-exclusive health states were modelled: health (without any *S*. *pneumoniae*-related diseases), bacteremia without pneumococcal pneumonia, bacteremia with pneumococcal pneumonia, meningitis, CAP caused by *S*. *pneumoniae*, neurological sequelae and death of or other than the related diseases (Fig 1). A Markov cycle for each stage was set at one year with a cohort timeframe of 15 years after being vaccinated. We assumed all the individuals who survived until the timeframe age have a life expectancy of the Japanese population [14]. Adverse effects associated with vaccination of PPSV–23 and PCV–13 were not considered, since they were mild or moderate in severity [15–17]. Considering that all transition states did not occur simultaneously at the end of each cycle, while in reality, most kinds of transitions typically occur gradually throughout a time interval (on average, half-way through), we implemented a half-cycle correction in estimating the incremental cost-effectiveness ratios (ICERs) of the programmes [18]. The half-cycle correction is implemented by using one-half of every state’s incremental reward in model’s initial and final reward.

### Outcomes estimation

Outcomes in terms of QALY were estimated by assigning transition probabilities and utility weights from literature. We estimated the 5-year age-specific incidence rates using the following: (1) annual IPD incidence rates among persons age 65 and over (2.41 per 100,000) [19], (2) IPD distribution by age [19], and (3) demographic data [10]. NPP annual incidence rates were estimated as incidence of CAP times proportion of *S*. *pneumonia*-caused CAP at 17.2% [20]. CAP incidence rates, 10.7 and 42.9 per 1000 person-years for persons who were aged 65–74 and aged ≥75, respectively, were from a 3-year prospective hospital-based surveillance [21]. Proportions of bacteremia with/without pneumococcal pneumonia, and meningitis among IPD cases were from the Infectious Agents Surveillance Report (IASR) [19]. Ubukata’s results of IPD case-fatality rates and proportion that results in neurological sequelae among IPD cases and NPP cases were used in the study [22]. NPP case-fatality rate was from Ishida et al.’s study, which reported the rate among patients with positive urinary antigen test of *S*. *pneumoniae*-related pneumonia [23]. Deaths of causes other than the above diseases were taken from the vital statistics [24]. Utility weights used to calculate QALY were assumed based on a study by Smith et al. [25]. Average lengths of hospital stay were from published government data [26]. All these data are shown in Table 1.

**Table 1:** Model inputs.

Distribution of population among adults aged 65 and older									
**Age**	**%**								[10]
65, 66, 67, 68, 69, 70, 71, 72, 73, 74,	6.9, 6.6, 4.1, 4.4, 5.3, 5.1, 5.2, 5.0, 4.5, 3.9,								
75, 76, 77, 78, 79, 80, 81, 82, 83, 84,	4.1, 4.2, 4.1, 3.8, 3.5, 3.4, 3.2, 3.0, 2.7, 2.5,								
85, 86, 87, 88, 89, 90, 91, 92, 93, 94,	2.3, 2.1, 1.9, 1.6, 1.3, 1.1, 0.9, 0.7, 0.6, 0.4,								
95, 96, 97, 98, 99,100+	0.3, 0.3, 0.2, 0.1, 0.1, 0.2								
	**Percentage of female among each age**								
	51.4, 51.5, 51.9, 52.4,52.7, 52.9,53.2, 53.4, 53.8, 54.2,								
	54.8, 55.3,55.9,56.7,57.6, 58.6, 59.3, 60.4,61.5,62.8								
	63.9, 65.4, 66.9, 69.2, 71.2 74.4, 76.3, 77.7, 78.4, 79.9								
	81.1, 81.6, 83.6,84.1,84.8, 87.3								
**Rate and proportions** ^**a**^	**65+**	**65–69**	**70–74**	**75–79**	**80–84**	**85–89**	**90–94**	**95+**	
Annual incidence rate of IPD (per 100,000 population)	2.4	1.8	1.8	2.5	2.7	4.2	4.4	4.2	[10, 19]
Bacteremia without pneumococcal pneumonia among IPD cases (%)	35.6	36.8	40.0	29.8	38.0	33.9	42.1	16.7	[19]
Bacteremia with pneumococcal pneumonia among IPD cases (%)	45.8	39.5	35.5	50.7	45.1	54.8	50.0	66.7	[18]
Meningitis among IPD cases (%)	18.6	23.6	24.5	19.6	16.8	11.3	7.9	16.7	[19]
Annual incidence rate of CAP (per 1,000 population)	10.7 (aged 65–74), 42.9 (age > = 75)								[21]
CAP caused by *S*. *pneumoniae* (%)	17.2								[20]
Bacteremia without pneumococcal pneumonia result in sequelae (%)	2.0								[22]
Bacteremia with pneumococcal pneumonia result in sequelae (%)	7.0								[22]
Meningitis result in sequelae (%)	30.0								[22]
Non-bacteremic pneumococcal pneumonia result in sequelae (%)	2.7								[22]
**Case-fatality rate (%)**												
Bacteremia without pneumococcal pneumonia	25.0								[22]
Bacteremia with pneumococcal pneumonia	30.5								[22]
Meningitis	14.9								[22]
Non-bacteremic pneumococcal pneumonia	1.9								[23]
Sequelae	5.0								[22]
**Serotypes covering of disease caused by *S*. *pneumococcus***									
Invasive pneumococcal diseases	PPSV–23: 60.0%		PCV–13: 46.0%						[19]
Non-bacteremic pneumococcal pneumonia	PPSV–23: 62.9%		PCV–13: 49.3%						[13]
**Utility weights**									[25]
Health	1								
Bacteremia without pneumococcal pneumonia	0.5								
Bacteremia with pneumococcal pneumonia	0.5								
Meningitis	0.4								
Pneumococcal pneumonia	0.5								
Sequelae	0.3								
Death	0								
**Average lengths of hospital stay (day)**	**65–69**	**70–74**	**75–79**	**80–84**	**85–89**	**90–94**	**95–99**	**100+**	
Bacteremia/pneumonia	12.3	13.1	14.1	14.9	15.6	16.1	16.3	16.2	[26]
Meningitis	24.5	26.3	28.3	29.9	31.2	32.1	32.6	32.4	[26]
**Treatment costs per case (¥)**									
Bacteremia/pneumonia	428,005	440,028	453,172	453,404	449,147	435,079	425,829	408,372	[26]
Meningitis	856,011	880,057	906,343	906,808	898,293	870,158	851,658	816,744	[26]
Sequelae (per case per year)	1,500,000								[27]
**Costs per vaccination (¥)**									
Cost per PPSV–23 shot	8,116								[28, 29]
Cost per PCV–13 shot	10,776								[28, 29]

^a^On Markov model, transition probabilities from health state A to health state B by ages were calculated as follows

From “Health” to “Bacteremia without pneumococcal pneumonia” = Annual incidence rate of IPD × Bacteremia without pneumococcal pneumonia among IPD cases

From “Health” to “Bacteremia with pneumococcal pneumonia” = Annual incidence rate of IPD × Bacteremia with pneumococcal pneumonia among IPD cases

From “Health” to “Meningitis” = Annual incidence rate of IPD × Meningitis among IPD cases

From “Health” to “Non-bacteremic pneumococcal pneumonia” = Annual incidence rate of CAP × CAP caused by *S*. *pneumoniae*

Vaccine effectiveness of PPSV–23 in reducing IPD incidence rate was cited from a Cochrane Review report [30]. Results from meta-analysis show that the use of PPSV–23 to prevent vaccine serotype IPD in adults of high-income countries, was at 82% (69%-90%), while its effectiveness in reducing non-IPD was inconsistent. We assumed that the effectiveness in reducing non-IPD to be 0% [30]. PCV–13 effectiveness in reducing vaccine-serotype IPD and non-invasive vaccine-type CAP, 75.0% and 45.0%, respectively, were from a randomised placebo-controlled trial study [31]. We assumed the effectiveness of both vaccines reduce by age of vaccination and by years after being vaccinated. Extent of reduction was proportional to the effectiveness used in Smith et al.’s study [25]. All these data are shown in Table 2. The vaccine serotypes causing IPD among elderly were 60.0% and 46.0% for PPSV–23 and PCV–13 [19], respectively, those for NPP were 62.7% and 49.3% [13] (Table 1).

**Table 2:** Data used to estimate vaccine effectiveness (VE) and VEs used in the model.

**Data used to estimate vaccine effectiveness (1–3)**												
1. Vaccine effectiveness of PPSV–23 and PCV–13 in preventing IPD used in Smith et. al's study [25] (%)												
	**PPSV–23**						**PCV–13**					
**years post**	**aged 65–79**			**aged 80 and over**			**aged 65 and over**					
**vaccination**	**base-case**	**low**	**high**	**base-case**	**low**	**high**	**base-case**	**low**	**high**			
1	80.0	60.0	90.0	67.0	20.0	85.0	85.0	60.0	95.0			
3	73.0	50.0	83.0	53.0	0	83.5	80.0	45.0	90.0			
5	58.0	30.5	80.0	32.0	0	75.0	70.0	30.0	87.0			
7	33.0	13.0	48.0	10.0	0	30.0	60.0	22.5	77.5			
10	0	0	10.0	0	0	10.0	50.0	15.0	68.0			
15	0	0	10.0	0	0	10.0	33.0	0	60.0			
2. Vaccine effectiveness of PPSV–23 in preventing vaccine type IPD (in high income countries) (%)												
	(based on Cochrane database of systemic review [30])											
	82	69	90									
3. Vaccine effectiveness of PCV–13 in preventing IPD and non-bacteremic CAP based on CApiTA study [31]) (%)												
	Reduced non-bacteremic vaccine type CAP				45.0							
	Reduced vaccine-type IPD				75.0							
**VE in preventing IPD used in current study (%)** (Based on 1, 2, and 3)												
	**PPSV–23**						**PCV–13**					
**years post**	**aged 65–79**			**aged 80 and over**			**aged 65–79**			**aged 80 and over**		
**vaccination**	**base-case**	**low**	**high**	**base-case**	**low**	**high**	**base-case**	**low**	**high**	**base-case**	**low**	**high**
1	82.0	69.0	90.0	68.7	23.0	85.0	75.0	52.9	83.8	62.8	17.6	79.2
3	74.8	57.5	83.0	54.3	0.0	83.5	70.6	39.7	79.4	51.2	13.2	75.0
5	59.5	35.1	80.0	32.8	0.0	75.0	61.8	26.5	76.8	34.1	8.8	72.5
7	33.8	15.0	48.0	10.3	0.0	30.0	52.9	19.9	68.4	16.0	6.6	64.6
10	0	0	10.0	0	0	10.0	44.1	13.2	60.0	0	0	56.7
15	0	0	10.0	0	0	10.0	29.1	0	52.9	0	0	50.0
**VE in preventing non-invasive vaccine type CAP used in current study (%)** (Based on 1, 2, and 3)												
	**PPSV–23**						**PCV–13**					
**years post**	**aged 65–79**			**aged 80 and over**			**aged 65–79**			**aged 80 and over**		
**vaccination**	**base-case**	**low**	**high**	**base-case**	**low**	**high**	**base-case**	**low**	**high**	**base-case**	**low**	**high**
1	-	-	-	-	-	-	45.0	31.8	50.3	37.7	26.6	42.1
3	-	-	-	-	-	-	42.4	23.8	47.6	30.7	17.3	34.6
5	-	-	-	-	-	-	37.1	15.9	46.1	20.4	8.8	25.4
7	-	-	-	-	-	-	31.8	11.9	41.0	9.6	3.6	12.4
10	-	-	-	-	-	-	26.5	7.9	36.0	0	0	0
15	-	-	-	-	-	-	17.5	0	31.8	0	0	0

### Costing

In this study, costs borne by government, municipalities, vaccinees, patients and third party payers were considered, while advertising costs borne by manufacturers were left unaccounted. It is obvious that when a new product enters a market, which was monopolised by the other product, the manufacturers of both products will invest a lot to compete for the share in the market. Since the decision maker, MHLW Vaccine Committee, was only interested in costs borne by the aforementioned payers, we omitted the inclusion of this cost. Non-direct medical costs related to the immunisation programme were not included, because the programme was built within the public health services routine [32]. The amount of direct payments to health care providers by these entities was estimated as costs. Cost items were identified along the decision tree and Markov model. We used the literature along with some assumptions to estimate necessary data.

Age-specific treatment costs of per case of bacteremia with/without pneumococcal pneumonia and pneumonia were estimated from Status Survey on Medical Care Benefits [26]. Cost per case of meningitis was assumed to be twice the cost per case of bacteremia, while cost of sequelae was assumed ¥1,500,000 (US$13,636) per case per year [27]. One vaccine shot was assumed at ¥8,116 (US$ 74: US$1 = ¥110) for PPSV–23 and ¥10,776 (US$98) for PCV–13, which were the sum of vaccine price (¥4,737 or US$43 for PPSV–23, ¥7,200 or US$65 for PCV–13) [28, 29], doctor fee and technical fee for PPSV–23 and PCV–13, respectively. All cost data are shown in Table 1.

### Discounting

Outcomes and costs were discounted at a rate of 3% [32].

### One-way sensitivity analyses and probabilistic analyses

We performed one-way sensitivity analyses on two pairs of comparisons. The first pair is ≥65 PPSV–23 strategy vs. current PPSV–23 strategy, which is to appraise the stability of ICERs with the assumptions made in our economic model, and to explore the impact of each variable relative to each other when the subsidy of the immunisation is limited to PPSV–23. The second pair is PCV–13 strategy vs. current PPSV–23 strategy, which is to appraise the same issues when PCV–13 is also subsidised by current immunisation programme. The lower limits and upper limits used in sensitivity analyses were ±50% for costs variables and ±20% for probabilities and utilities. We also conducted two sets of 1000 Monte Carlo simulations on ≥65 PPSV–23 strategy and 65–80 PPSV–23 strategy vs. current PPSV–23 strategy, i.e., probabilistic analyses, for which all data were assumed to have an equilateral triangle distribution corresponding to the range tested in one way sensitivity analyses. Triangular distribution was used because of the insufficiency of information about distributions. This distribution has been theoretically proven as both simple and efficient, which can serve as a proxy for beta or other distributions in risk analysis [33–35].

## Results

### Costs, effectiveness, and cost-effectiveness of alternative PPSV–23 single-dose immunisation strategies

Compared to current PPSV–23 strategy, the incremental cost and incremental effectiveness per person for ≥65 PPSV–23 strategy were ¥216 (US$2) and 0.00004 QALYs; estimated ICER was ¥5,025,000 (US$45,682) per QALY gained. For 65–80 PPSV–23 strategy, incremental cost and incremental effectiveness were both negative values, which indicated that 60–80 PPSV–23 strategy cost less but also gained less than current PPSV–23 strategy (Table 3).

**Table 3:** Cost, effectiveness and incremental cost-effectiveness ratio (vs. current scenario) by using PPSV–23 only.

	Vaccine cost per person	Treatment cost per person	Total cost per person	Effectiveness per person	Incremental cost	Incremental effectiveness	ICER* = (5)/(6)
	¥	¥	¥	QALY	¥	QALY	
	(1)	(2)	(3) = (1)+(2)	(4)	(5)	(6)	
Current strategy	3,860	20,456	24,316	14.31480	-	-	-
65–80 strategy	2,259	20,460	22,719	14.31480	-1,597	-0.00001	cost less, gain less
≥65 strategy	4,091	20,441	24,532	14.31485	216	0.00004	5025,000

*ICER: incremental cost-effectiveness ratio (¥/QALY gained). All ICERs were rounded to the nearest thousand.

### Costs, effectiveness, and cost-effectiveness of including PCV–13 in the list of single-dose subsidy

Among 10 scenarios with different PCV–13 diffusion levels, scenarios with higher PCV–13 diffusion level resulted in larger vaccine cost, while it saved more treatment costs and gained more QALYs compared to current scenario. Reduced treatment costs did not offset vaccination cost, which means gained more QALYs with more costs. ICERs were ¥378,000 (US$3,436) per QALY gained regardless of PCV–13 diffusion level (Table 4).

**Table 4:** Cost, effectiveness and incremental cost-effectiveness ratio of different diffusion levels of PCV–13 (vs. current PPSV–23 strategy).

Diffusion level of PCV13 vs. PPSV–23	Vaccine cost per person	Treatment cost per person	Total cost per person	Effectiveness per person	Incremental cost	Incremental effectiveness	ICER* = (5)/(6)
	¥	¥	¥	QALY	¥	QALY	
	(1)	(2)	(3) = (1)+(2)	(4)	(5)	(6)	
0% vs. 100% (Current strategy)	3,860	20,456	24,316	14.31480	-	-	-
10% vs. 90%	3,987	20,350	24,337	14.31486	21	0.00006	378,000
20% vs. 80%	4,113	20,245	24,358	14.31491	42	0.00011	378,000
30% vs. 70%	4,240	20,140	24,380	14.31497	64	0.00017	378,000
40% vs. 60%	4,366	20,035	24,401	14.31503	85	0.00022	378,000
50% vs. 50%	4,493	19,929	24,422	14.31508	106	0.00028	378,000
60% vs. 40%	4,619	19,824	24,443	14.31514	127	0.00034	378,000
70% vs. 30%	4,746	19,719	24,464	14.31520	149	0.00039	378,000
80% vs. 20%	4,872	19,613	24,486	14.31525	170	0.00045	378,000
90% vs. 10%	4,999	19,508	24,507	14.31531	191	0.00051	378,000

*ICER: incremental cost-effectiveness ratio (¥/QALY gained). All ICERs were rounded to the nearest thousand.

## Results of one-way sensitivity analyses and probabilistic analyses

**Fig 2:**
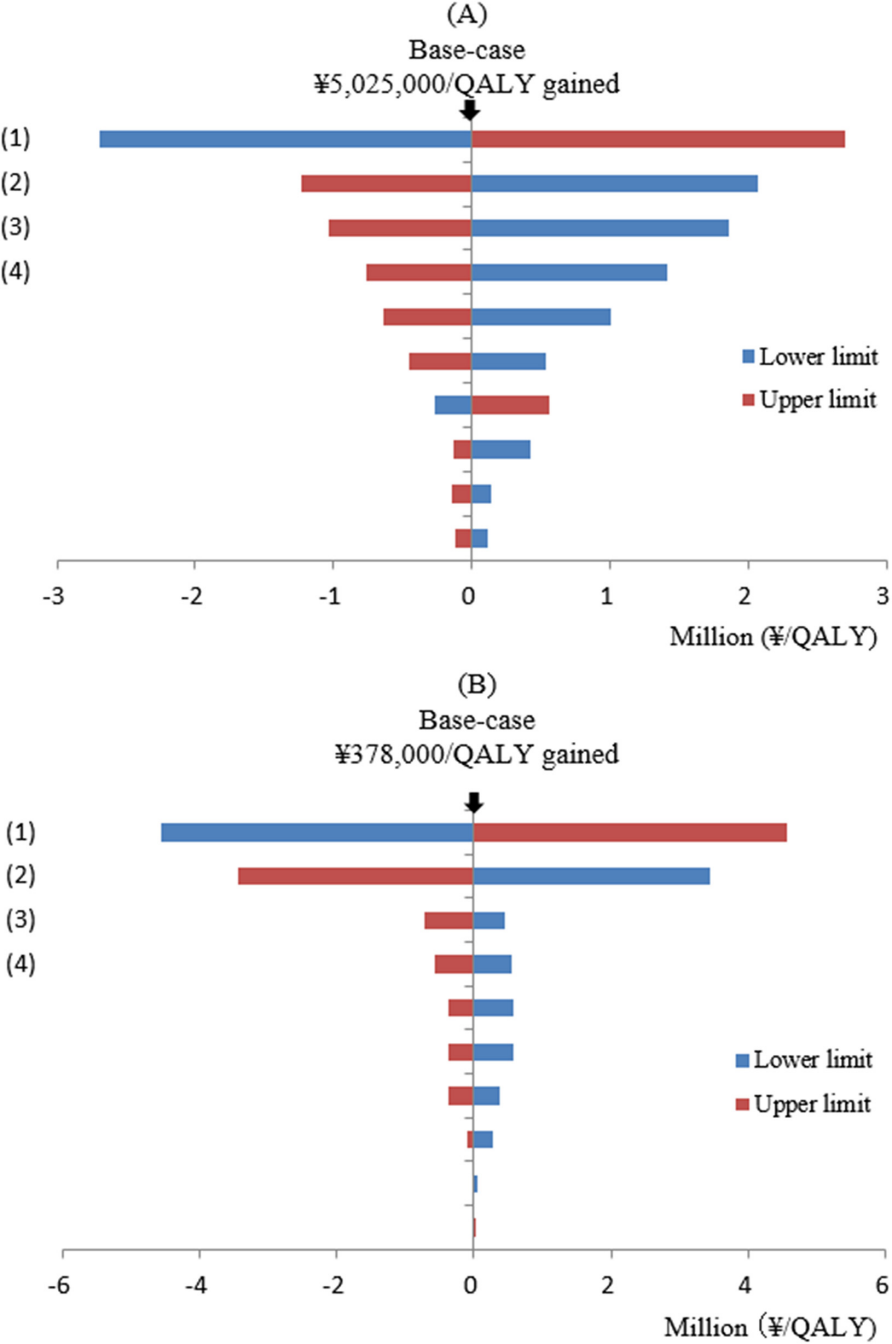
Results of one-way sensitivity analyses. (A) ≥65 PPSV–23 strategy vs. current PPSV–23 strategy. (B) PCV–13 strategy vs. current PPSV–23 strategy. In Fig 2A: (1) Cost per shot of PPSV–23, (2) Annual incidence rate of IPD, (3) Vaccine effectiveness of PPSV–23 in reducing IPD incidence rate, (4) Percentage of vaccine serotype causing IPD. In Fig 2-B: (1) Cost per shot of PCV–13. (2) Cost per shot of PPSV–23, (3) Vaccine effectiveness of PCV–13 in preventing noninvasive vaccine type CAP, (4) Treatment cost per *S*. *pneumoniae*-related case.

**Fig 3:**
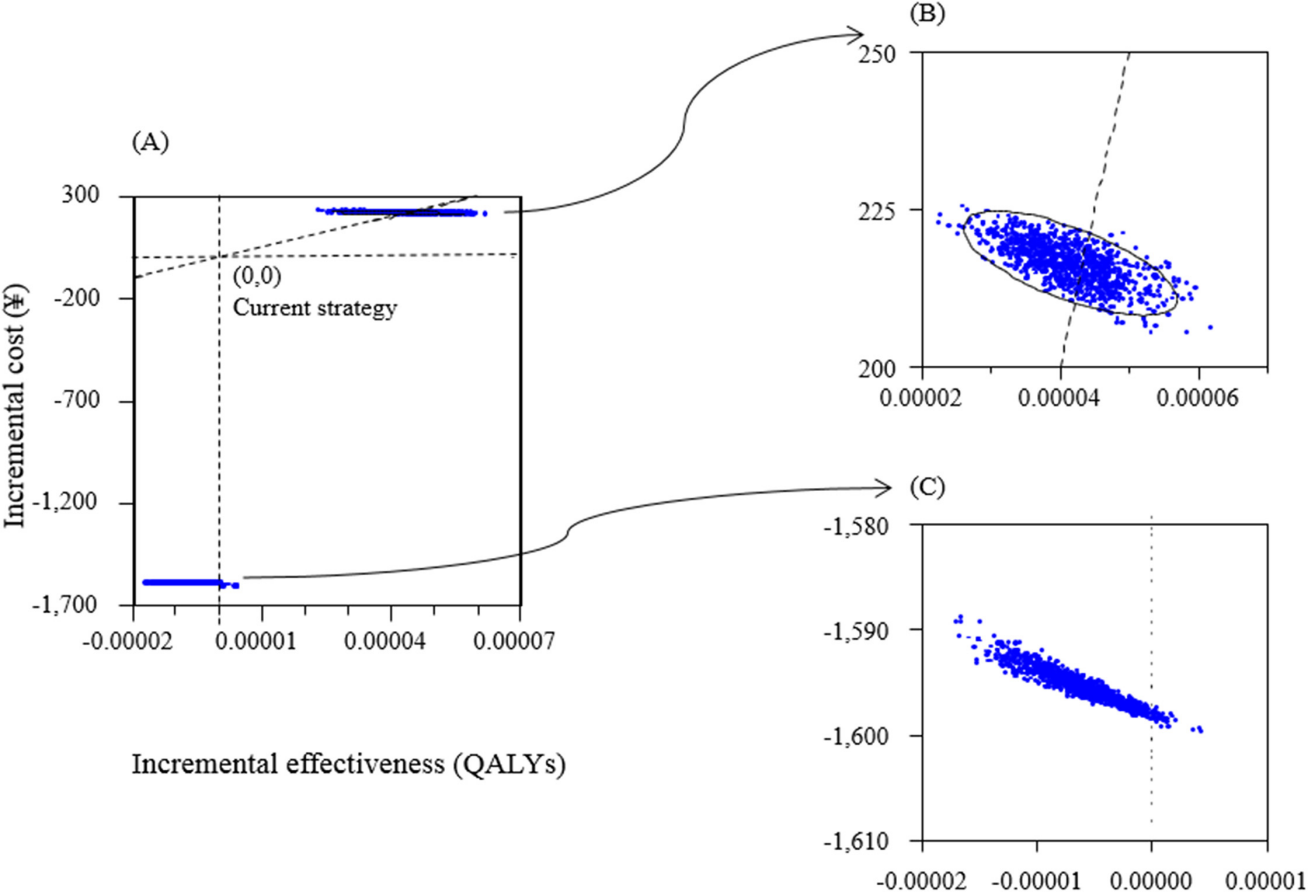
Results of probabilistic sensitivity analyses. (A) Scatter plot of incremental cost and incremental effectiveness per person of ≥65 PPSV–23 strategy vs. current PPSV–23 strategy and 65–80 PPSV–23 strategy vs. current PPSV–23 strategy. (B) Enlarged view of ≥65 PPSV–23 strategy vs. current PPSV–23 strategy. (C) Enlarged view of 65–80 PPSV–23 strategy vs. current PPSV–23 strategy.

**Fig 4:**
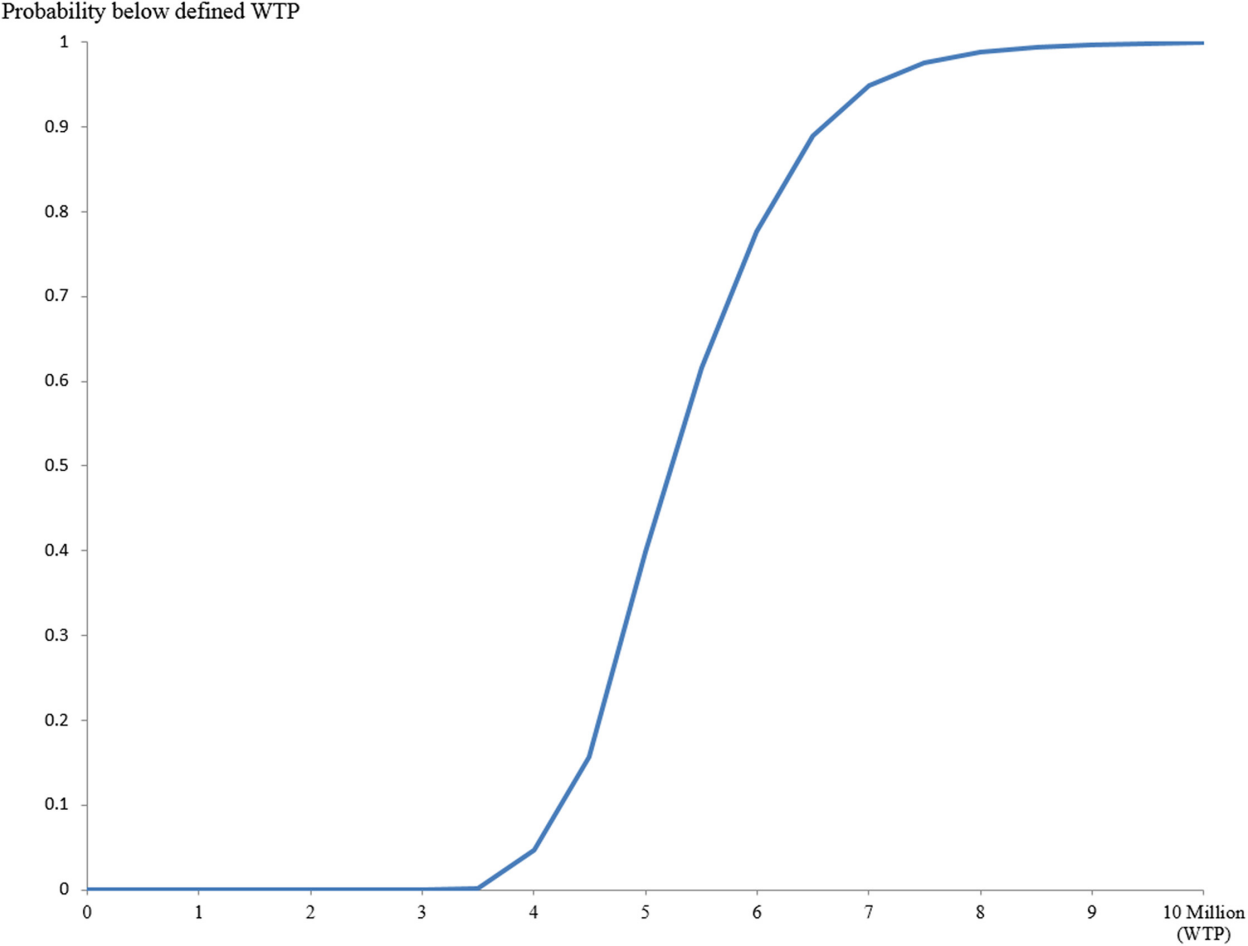
Cost-effectiveness acceptability curve (CEAC) of ≥65 PPSV–23 strategy vs. current PPSV–23 strategy.

In one-way sensitivity analyses of ≥65 PPSV–23 strategy vs. current PPSV–23 strategy, the variables which were found to increase/decrease ICER more than ¥1,000,000 (US$9,091) are as follows: (1) cost of per vaccine shot, (2) IPD incidence rate, (3) vaccine effectiveness of PPSV–23 in reducing IPD incidence rate, and (4) percentage of vaccine serotype causing IPD (Fig 2A). In PCV–13 strategy vs. current PPSV–23 strategy, only costs per shot of PCV–13 and per shot of PPSV–23 were found to produce large changes in ICERs (Fig 2B). Figs 3 and 4 show the results of probabilistic analyses. Each dot on Fig 3 represents the incremental cost and effect obtained from one simulation following the random draw of model parameters from distribution. The cost-effectiveness acceptability curve (CEAC) shows that in ≥65 PPSV–23 strategy vs. current PPSV–23 strategy, among 1000 ICERs produced by Monte Carlo simulations, 61.5% are under ¥5,500,000 (US$50,000) per QALY and 100% are under ¥10,000,000 (US$90,910) per QALY (Fig 4).

## Discussion

This study intends to address the following objectives: (1) to evaluate the efficiency of alternative PPSV–23 immunisation strategies compared to current PPSV–23 strategy, and (2) to investigate the cost-effectiveness of the inclusion of PCV–13 in the list of single-dose current pneumococcal vaccine national immunisation programme.

Compared to the current PPSV–23 strategy, incremental cost and incremental effectiveness of 65–80 PPSV–23 strategy were both negative, which means that switching current strategy to 65–80 strategy was found to gain less QALYs than current PPSV–23 strategy (this outcome will not be considered in decision-making). Switching current PPSV–23 strategy to ≥65 PPSV–23 strategy was found to be favourable (ICER at ¥5,025,000 or US$45,682 per QALY gained) compared to either of the suggested criterion by WHO of three times GDP (around ¥11,000,000 or US$113,636 per QALY gained in Japan) [36], or by Shiroiwa at ¥5,000,000 (US$45,455) per QALY gained [37]. Moreover, the result of probabilistic sensitivity analyses on switching current PPSV–23 strategy to ≥65 PPSV–23 strategy, ICER to be under ¥5,500,000 (US$50,000) per QALY is 61.5% and is 100% to be under ¥10,000,000 (US$90,910) per QALY gained, is deemed to be cost-effective.

We compared 10 scenarios (with 10 PCV–13 diffusion levels) to current PPSV–23 strategy. Results showed that PCV–13 inclusion in the subsidy list has value for money (ICER = ¥378,000 or US$3,436 per QALY gained, regardless of PCV–13 diffusion level).

Since there are only a few variables which will induce the ICER to go beyond ¥1,000,000 (US$9,091) per QALY gained, we consider our results to be robust. In comparing ≥65 PPSV23 strategy with current PPSV23 strategy, the top four variables which have the biggest impact on ICER were cost per vaccine shot, IPD incidence rate, vaccine effectiveness, and percentage of vaccine serotype causing IPD. On the other hand, in comparing PCV–13 strategy with current PPSV–23, only cost per vaccine shot will change the ICER larger than ¥1,000,000 (US$9,091) per QALY.

This study has several limitations. In Japan, before the national immunisation programme was launched, some municipalities already provided subsidies to the elderly for single shot PPSV–23 from 2001 to September 2014 with a vaccine coverage of about 25% [3]. We didn’t incorporate the already-vaccinated group in our model since the efficiencies of the programmes were determined by incremental difference of costs and QALYs between the comparator and alternatives; its influence to the results should be limited. Due to insufficient data of the municipality-led PPSV–23 immunisation programmes for the elderly, we decided not incorporate this into the model. Considering that more elderly will uptake vaccine through these extra programmes, the strategy will move from current PPSV–23 strategy towards ≥ 65 PPSV–23 strategy and these results will be useful for those municipalities. We assumed that in both 65–80 PPSV–23 strategy and ≥ 65 PPSV–23 strategy, the eligible persons will uptake vaccine in the first year. Since the incidence rates of pneumococcal diseases and the vaccine effectiveness varies with age, it was difficult to predict how the results will change. Based on previous study [38], if an eligible person uptakes vaccine around 70–75 years old, it would bring more favourable results to both 65–80 PPSV–23 strategy and ≥65 PPSV–23 strategy. We didn’t incorporate the herd effect of PCV–7 or PCV–13 immunisation programmes among children, which was likely to indirectly protect the elderly and thus potentially reducing the efficiencies of the immunisation programme using both vaccines. We deferred its incorporation as it might pose some bias to the result. Though several studies do provide some evidence for the existence of such an effect, further evidence is required before definite interpretations can be made. The decreasing vaccine-serotype IPD and non-invasive vaccine-type CAP cases due to serotype replacement during the 15-year cohort time were not incorporated. The replacement occurred in Japan after the launching of children’s PCV immunisation programme has decreased the vaccine-serotype IPD among adults from 85% (PPSV–23) and 61% (PCV–7) in 2007 [39] to 60% (PPSV–23) and 40% (PCV–13) in 2013, respectively [19]. The advertising costs borne by manufacturers were left unaccounted. Incorporating these might bring more unfavourable results.

Several studies have compared the cost-effectiveness of the use of either PPSV–23 or PCV–13 or substitution of PPSV–23 with PCV–13 among elderly. Different studies has shown that both PPSV–23 and PCV–13 were cost-effective, and PCV–13 has high value for money than PPSV–23 [25, 40–44]. ICERs of all three PPSV–23 strategies vs. do-nothing in our study were too high to conclude that PPSV–23 immunisation programmes for the elderly were cost-effective (data in S1 Table), which was inconsistent with the results of previous studies. Inconsistencies were due to low incidence rates, low fatality rates, and low proportions of sequelae caused by *S*. *pneumoniae* in our study compared to those in previous studies. All 10 scenarios with different levels of share of PCV–13 have favourable ICERs but were not cost-saving compared to current strategy, which was inconsistent with the results of previous studies. The inconsistency observed was due to high vaccination cost.

Regardless of these limitations, we make efforts on literature survey to find out the available data of epidemiology and prognosis of relevant diseases which were considered to reflect the current situation of diseases caused by *S*. *pneumoniae* in Japan. We believe our results will provide useful results to policymakers.

## Conclusion

Results of our analyses indicate switching the current strategy to ≥65 scenario or including PCV–13 into the list for single-dose subsidy to the elderly in Japan has value for money. A further budget impact analysis is awaited for well-informed policymakers.

## Supporting Information

S1 Table Cost, effectiveness and incremental cost-effectiveness ratio (vs. do-nothing) by using PPSV–23 only. (DOCX)
