# Supplementary material for: Economic Evaluation of Immunisation Programme of 23-Valent Pneumococcal Polysaccharide Vaccine and the Inclusion of 13-Valent Pneumococcal Conjugate Vaccine in the List for Single-Dose Subsidy to the Elderly in Japan
Source: PLoS One. 2015 Oct 7;10(10):e0139140. doi: 10.1371/journal.pone.0139140 (PMC4596483; doi:10.1371/journal.pone.0139140)
Supplement: S1 Table — (DOCX) [file pone.0139140.s001.docx]

S1 Table. Cost, effectiveness and incremental cost-effectiveness ratio (vs. do-nothing) by using PPSV-23 only.

|  | Vaccine cost  per person | Treatment cost  per person | Total cost  per person | Effectiveness  per person | Incremental cost | Incremental effectiveness | ICER* |
| --- | --- | --- | --- | --- | --- | --- | --- |
|  | ¥ | ¥ | ¥ | QALY | ¥ | QALY | =(5)/(6) |
|  | (1) | (2) | (3)=(1)+(2) | (4) | (5) | (6) |  |
| Do nothing | 0 | 20,496 | 20,496 | 14.31471 | comparator | comparator | - |
| Current strategy | 3,860 | 20,456 | 24,316 | 14.31480 | 3,820 | 0.00009 | 41,975,000 |
| 65-80 strategy | 2,259 | 20,460 | 22,719 | 14..31480 | 2,222 | 0.00008 | 26,147,000 |
| ≥65 strategy | 4,091 | 20,441 | 24,532 | 14.31485 | 4,036 | 0.00013 | 30,118,000 |
| *ICER: incremental cost-effectiveness ratio (¥/QALY gained). All ICERs are rounded to the nearest thousand. | | | | | | | |
